# Quality circles to identify barriers, facilitating factors, and solutions for high-quality primary care for asylum seekers

**DOI:** 10.3399/bjgpopen17X101133

**Published:** 2017-10-04

**Authors:** Cornelia Straßner, Sandra Claudia Gewalt, Peta Becker von Rose, Detlef Lorenzen, Joachim Szecsenyi, Kayvan Bozorgmehr

**Affiliations:** 1 GP & Researcher, Department of General Practice and Health Services Research, University Hospital Heidelberg, Heidelberg, Germany; 2 Researcher, Department of General Practice and Health Services Research, University Hospital Heidelberg, Heidelberg, Germany; 3 GP, Werkstatt Gesundheit e.V, Heidelberg, Germany; 4 GP & Head of Department of General Practice and Health Services Research, University Hospital Heidelberg, Heidelberg, Germany; 5 Senior Researcher, Department of General Practice and Health Services Research, University Hospital Heidelberg, Heidelberg, Germany

**Keywords:** quality circle, asylum seeker, primary care, reception centre

## Background

In 2015 Germany received more than 476 600 asylum applications.^1^ Incoming asylum seekers are accommodated in reception centres (RCs) for up to 6 months before they are dispersed to other federal states or districts. Due to the high immigration since the end of 2014, many federal states established new RCs to expand their capacities in hosting asylum seekers. Baden Württemberg, for example, one of the largest federal states receiving about 13% of incoming asylum seekers, expanded its capacity from one RC up until 2014 to five RCs thereafter. Since there are no nationwide standards in place, healthcare provision in RCs is highly heterogeneously organised and fragmented.^2^ In Heidelberg, former barracks of the US army were reorganised as an RC in August 2015 and hosted about 6500 asylum seekers. The concentration of asylum seekers in the RC, linked with insufficient provision of primary health care, led to an unmanageable number of consultations in emergency departments of nearby hospitals. Asylum seekers have specific healthcare needs due to exposure to pre-, peri-, and postmigration health risks. These include traumatic events,^3^ endemic infectious diseases in the countries of origin or transit,^4^ and chronic conditions which may have been exacerbated during the migration process. They are also at higher risk of developing psychological distress^5^ and acquiring infectious diseases in the host country due to mass accommodation.^6^

To address both the shortcomings in primary care provision and the special needs, a walk-in clinic jointly led by the university hospital, the public health services, and the local physicians’ association was established in the RC with funds from the state government and the university hospital. The clinic provides general medicine as well as gynecological, paediatric, and psychiatric and psychosomatic health care.

The aim of this article is to report challenges and solutions of establishing high-quality primary health care for asylum seekers and meeting their specific needs in the particular setting of a large RC.

Although other countries may not have similar RCs, the situation may change as the contemporary migration flows are very dynamic. Thus, this report may be useful for GPs to gain an insight into possible ways of managing these highly vulnerable people whose complex health needs often present a challenge in conventional healthcare settings.

## Analysis of current care

After a negotiation and planning period of 8 months, medical services were initiated in February 2016. In July 2016 a group discussion among 24 healthcare professionals working in the clinic was organised to conduct a situation analysis and to identify barriers and solutions for the provision of high-quality care. Emerging issues could be assigned to 10 major themes (Box 1). The ***cooperation*** of different professions in the clinic was seen as advantage but due to a high turnover of staff assuring continuity of care was challenging. In this context, the need for improved ***documentation*** of medical data was raised. ***Medical treatment*** was perceived more difficult than usual due to high numbers of patients with special needs (for example, drug-addicted patients or patients with infectious diseases such as tuberculosis or hepatitis) and lack of country-specific diagnostic algorithms. A lack of ***resources***, especially of assistants, interpreters, and medical supplies, affected the provision of care. Yet the general infrastructure of the clinic including the in-door-pharmacy was considered an advantage. ***Legal aspects*** were also mentioned as aggravating factors: The German Asylum Seekers Benefits Act limits the provision of care to acute and painful conditions, maternity care services, preventive medical checkups, vaccinations, and so called 'indispensable services'.^7^ This vague definition created uncertainties among providers about the scope of care provided in the clinic: acute and emergency care or continuous primary and specialised care. Thus, the scope of care provided was highly dependent on the personal ***attitude*** of the individual physician. However, the strong commitment of all staff who perceived their tasks as being meaningful was considered a facilitating factor.

**Box 1.** Barriers, enablers and strategies for assuring high-quality care for asylum seekers in a German reception centre

Theme	Barriers	Facilitators	Solutions
**Cooperation**	Lack of exchange of relevant information between providers	Trustful interprofessional cooperation of university and local resident physicians in one clinic	Consequent use of a previously introduced paper-based patient health record^11^
**Documentation**	Loss of information due to insufficient documentation/poor readability Dual documentation in two record systems (one of the university hospital, one of the local resident doctors) Unclear how to archive medical and laboratory results	Availability of a previously introduced paper-based personal health record^11^	Clarification with the university hospital’s administration to only use one documentation software Design of a pattern to standardise documentation in the software Software training for resident physicians was performed Installation of a software module to import laboratory results and training of staff how to use it
**Medical treatment**	Unclear how to handle patients with psychotropic dependency Shortcomings in assuring follow-up visits and continuity of care Lack of guidelines or standards for frequent reasons of consultation and infectious diseases Lack of time to address psychosocial causes of symptoms	Interprofessional, interdisciplinary team	Composition of a detoxification contract in various languages that all patients with psychotropic dependency have to sign Exchange with staff of advisory services for asylum seekers for social and legal issues during a quality circle meeting Guideline for diagnostics of infectious diseases will be elaborated in cooperation with the Department of Tropical Medicine
**Human resources**	Lack of interpreters Lack of support staff for medical and administrative tasks	Support by medical students	Employment of interpreters for frequent languages Employment of nurses
**Material resources**	Lack of drug supplies and vaccinations Shortage and lacking standardisation of necessary equipment Lack of well-functioning soft- and hardware (such as record system and printers)	Existence of an in-house pharmacy Adequate facility	Design of a checklist and implementation of a daily tour through the facility by a designated person to assure availability of equipment
**Politics and legislation**	Uncertainties among healthcare professionals about the scope of care covered by Asylum Seekers’ Benefits Act Uncertainties related to issuing medical certificates with relevance for the asylum procedure		Invitation of an advocate specialised in asylum law is planned
**Motivation/attitudes**	Unclear self-concept of the walk-in clinic: emergency department versus primary care practice	Work is considered a meaningful task Work is considered interesting because of wide range of cases High level of commitment from all staff	
**Patient behaviour**	High number of family members attending consultations Cultural differences in communication and behaviour	Thankful patients	Workshop on cultural competence was offered
**Healthcare providers’ behaviour**	Lack of discretion (staff comes in without knocking)		Room divider was bought
**Organisational processes**	Registration: lack of triage mechanisms to identify patients with acute conditions Registration: previous healthcare provision is not considered leading to redundancies in diagnostics and channeling to wrong specialisation Emergency case is not complete		Checklist for content of emergency case and a responsible person for regular controls were defined

## Solutions to address barriers and facilitating factors

The findings of the situation analysis led to the joint agreement to introduce a quality circle (QC). QCs can be defined as autonomous peer groups of healthcare professionals who meet on a regular basis and aim at assessment and improvement of quality of care in their own practices.^8^ They are characterised by a result-oriented approach identifying quality problems and strategies to address these problems.^9^ QCs have become a globally recognised instrument for quality improvement in Europe^8,9^ and are partly even obligatory for GPs in South Germany.

Two measures were undertaken to support the implementation of a regular and effective QC for clinic staff: the participants themselves decided on the frequency, the course and content of the meetings (bottom-up approach). The QC was accredited and the participants received credits for their continuous medical education.

Four QC meetings were held so far in intervals of 6–8 weeks. Each took approximately 2 hours and was marked by vivid discussions. With, on average, 24 participants from different professions and disciplines participation was continuously high. Although we did not conduct a systematic evaluation of the QC, we monitored the target achievements as this is part of the QC concept. Several solutions have been elaborated and implemented so far (Box 1). Measures on an organisational level such as creating checklists for the equipment and standardising procedures were implemented successfully. Difficulties were faced when barriers on a legal, political, or national level had to be addressed; for example, to create guidelines for frequent reasons for counselling in accordance with national guidelines and the entitlements covered by the Asylum Seekers’ Benefits Act.

## Discussion

When establishing healthcare services for asylum seekers many quality issues —﻿ partly similar, partly distinct from usual care —﻿ arise. Our experiences show that QCs, which have proved a suitable instrument for quality improvement in German primary care over years, are also helpful to foster quality improvements in special settings such as RCs for asylum seekers. However, structural barriers such as legal restrictions, financial limitations, or a lack of national clinical guidelines for the specific setting of RCs limit the full potential of QCs to improve care. Beside local initiatives national endeavours are necessary to assure high standards of health care for asylum seekers and to avoid harm, as the medical code of ethics demands. Health care for asylum seekers in Germany is currently characterised by an overwhelming heterogeneity due to decentralised organisation and responsibilities.^1,10^ We argue that measures to assure high quality standards should be enforced in healthcare services for asylum seekers just as in regular care. QCs may be an effective instrument for quality improvement beyond conventional audit approaches. We recommend to establish QCs in respective institutions.
